# Pre-steady-state Kinetic Analysis of a Family D DNA Polymerase from *Thermococcus* sp. 9°N Reveals Mechanisms for Archaeal Genomic Replication and Maintenance*

**DOI:** 10.1074/jbc.M115.662841

**Published:** 2015-07-09

**Authors:** Kelly M. Schermerhorn, Andrew F. Gardner

**Affiliations:** From New England Biolabs, Inc., Ipswich, Massachusetts 01938

**Keywords:** archaea, DNA polymerase, DNA replication, enzyme kinetics, kinetics, pre-steady-state kinetics, exonuclease

## Abstract

Family D DNA polymerases (polDs) have been implicated as the major replicative polymerase in archaea, excluding the Crenarchaeota branch, and bear little sequence homology to other DNA polymerase families. Here we report a detailed kinetic analysis of nucleotide incorporation and exonuclease activity for a Family D DNA polymerase from *Thermococcus* sp. 9°N. Pre-steady-state single-turnover nucleotide incorporation assays were performed to obtain the kinetic parameters, *k*_pol_ and *K_d_*, for correct nucleotide incorporation, incorrect nucleotide incorporation, and ribonucleotide incorporation by exonuclease-deficient polD. Correct nucleotide incorporation kinetics revealed a relatively slow maximal rate of polymerization (*k*_pol_ ∼2.5 s^−1^) and especially tight nucleotide binding (*K_d_*_(dNTP)_ ∼1.7 μm), compared with DNA polymerases from Families A, B, C, X, and Y. Furthermore, pre-steady-state nucleotide incorporation assays revealed that polD prevents the incorporation of incorrect nucleotides and ribonucleotides primarily through reduced nucleotide binding affinity. Pre-steady-state single-turnover assays on wild-type 9°N polD were used to examine 3′-5′ exonuclease hydrolysis activity in the presence of Mg^2+^ and Mn^2+^. Interestingly, substituting Mn^2+^ for Mg^2+^ accelerated hydrolysis rates >40-fold (*k*_exo_ ≥110 s^−1^ *versus* ≥2.5 s^−1^). Preference for Mn^2+^ over Mg^2+^ in exonuclease hydrolysis activity is a property unique to the polD family. The kinetic assays performed in this work provide critical insight into the mechanisms that polD employs to accurately and efficiently replicate the archaeal genome. Furthermore, despite the unique properties of polD, this work suggests that a conserved polymerase kinetic pathway is present in all known DNA polymerase families.

## Teaser

**Background:** Family D DNA polymerase (polD) is important for replication in most archaea, excluding Crenarchaeota.

**Results:** We report a detailed kinetic characterization of polD nucleotide incorporation, mismatch discrimination, and 3′-5′ exonuclease hydrolysis.

**Conclusion:** Despite evolutionary divergence, polD kinetic pathways share similarities to other DNA polymerase families.

**Significance:** This work contributes to unifying our understanding of DNA polymerase function.

## Introduction

DNA polymerases play central roles in genome replication, maintenance, and repair and are therefore critical for genome integrity. Consequently, DNA polymerases have been the subject of extensive and widespread research for over 60 years (1). Multiple sequence alignment studies have classified DNA polymerases into seven different families: A, B, C, D, X, Y, and reverse transcriptases (RTs) (2–4). Kinetic studies of Family A, B, C, X, Y, and RT DNA polymerases have proven to be a powerful tool in understanding polymerase function (5–7). Importantly, such studies reveal the kinetic basis of nucleotide selection and mismatch discrimination mechanisms, offering critical insight into how DNA polymerases accurately and efficiently synthesize and maintain genomes.

Although the majority of DNA polymerase families have been well characterized, studies of Family D DNA polymerases (polDs),^2^ found in all known archaea (excluding the Crenarchaea branch), have been limited (8). Previous polD characterization studies have revealed that this polymerase is heterodimeric, composed of a large polymerase subunit and small 3′-5′ exonuclease proofreading subunit (9–11). The activities of the two subunits are co-dependent, and the presence of both is required for activity of either unit, a feature unique to the polD family (12, 13). Gene deletion studies in *Thermococcus kodakarensis* and *Methanococcus maripaludis* suggest that polD is essential for cell viability and indicate that polD may be the major replicative polymerase responsible for leading and lagging strand synthesis in these organisms (14, 15). Furthermore, polD forms complexes with many replisome components, including the minichromosome maintenance helicase, proliferating cell nuclear antigen processivity factor, and DNA ligase, providing further support for the belief that polD is a replicative polymerase in Archaea (15–18). It is still unclear what specific role(s) polD plays in archaeal genome replication and which roles are played by other DNA polymerases. For example, efficient Okazaki fragment maturation is dependent on a Family B DNA polymerase (19).

The biochemical properties of a well expressed polD from *Thermococcus* sp. 9°N have been characterized in our laboratory (20). In this work, a qualitative assessment of 9°N polD 3′-5′ exonuclease activity showed a dependence on Mg^2+^ or Mn^2+^ for catalytic activity, with a preference for Mn^2+^ (20). Despite its 3′-5′ exonuclease, recombinant polD has relatively low fidelity compared with most other characterized DNA polymerases (20).

To gain further insight into this unique multisubunit polymerase, we have performed a detailed kinetic characterization of the polymerase activity of an exonuclease-deficient mutant of polD, including kinetics of correct nucleotide incorporation and pyrophosphorolysis as well as incorrect nucleotide and ribonucleotide discrimination kinetics. Furthermore, we kinetically characterized the 3′-5′ exonuclease activity of wild type (WT) polD at 60 °C in the presence of Mn^2+^ and Mg^2+^. This detailed kinetic analysis performed on a Family D DNA polymerase provides insight into polymerase and exonuclease reaction pathways and suggests mechanisms for archaeal genome maintenance.

## Experimental Procedures

### 

#### 

##### Enzymes, Oligonucleotides, and Reagents

WT *Thermococcus* sp. 9°N polD and an exonuclease-deficient polD mutant (H554A) (abbreviated as polD^−^ for clarity) were expressed and purified as described previously (20). polD small and large subunits form a heterodimeric complex in a ∼1:1 molar ratio, as determined by SDS-PAGE gel analysis and quantitation by densitometry (data not shown). Oligonucleotides used in this study were purchased from Integrated DNA Technologies (Coralville, IA) (Table 1). For oligonucleotide detection, the primer strand was 5′-labeled with a 5-carboxyfluorescein (FAM) fluorophore (Integrated DNA Technologies). Buffers used in this study were from New England Biolabs (Ipswich, MA).

##### polD^−^ Steady-state Single Nucleotide Incorporation Assay

A steady-state kinetic assay was performed to determine whether polD− followed burst kinetics and to calculate an active enzyme concentration. The primer·template used to monitor steady-state kinetics was prepared by annealing the 50-mer 5′-FAM primer (10 μm) (Table 1) to the 62-mer “C Template-1” (15 μm) (Table 1) in 1× ThermoPol buffer (20 mm Tris-HCl, 10 mm (NH_4_)_2_SO_4_, 10 mm KCl, 2 mm MgSO_4_, 0.1% Triton X-100, pH 8.8, at 25 °C) by heating to 95 °C for 3 min, followed by cooling to room temperature. A 250-μl polD^−^/DNA aliquot was prepared by mixing ThermoPol buffer (1× final concentration), primer·template DNA (80 nm final concentration), and polD^−^ (20 nm final concentration) to ensure a 4-fold excess of DNA to polD^−^. A second 250-μl aliquot was prepared by mixing ThermoPol buffer (1× final concentration) and dGTP (200 μm final concentration). Using a rapid chemical quench instrument (RQF) (KinTek Corp., Snow Shoe, PA) set to 62.5 °C with a circulating water bath to achieve a final 60 °C reaction temperature, the polD^−^/DNA and dGTP solutions were rapidly mixed from 0.07 to 10 s and quenched with 50 mm EDTA. After mixing of equal volumes of polD^−^/DNA and dGTP solutions by the RQF, the final reaction concentrations were 40 nm DNA, 10 nm polD^−^, and 100 μm dGTP in 1× ThermoPol buffer. A negative control reaction was performed in which the dGTP aliquot was replaced with 1× ThermoPol buffer and reacted in the RQF with polD^−^/DNA for 10 s. To ensure that the DNA substrate (40 nm) was in molar excess to satisfy steady-state requirements, control experiments were performed with a fixed polD^−^ concentration (10 nm final concentration) and varying DNA concentrations (10–80 nm final concentration) as described above (data not shown). Furthermore, the dependence of burst amplitude on enzyme amount was confirmed (data not shown). Reaction products were separated by capillary electrophoresis using a 3730xl Genetic Analyzer (Applied Biosystems), and fluorescent peaks were analyzed using Peak Scanner software version 1.0 (Applied Biosystems). The concentration of product (51 nt of DNA with a FAM label) was graphed as a function of time, and the data were fit to burst Equation 1 using the nonlinear regression program Kaleidagraph (Synergy Software).

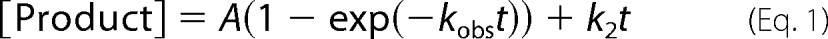
 From the fit, one can extract the active enzyme concentration (*A*), the initial rate of product formation (*k*_obs_), and steady-state turnover rate (*k*_ss_), which is obtained by dividing *k*_2_ by *A*. All kinetic assays described in this work were performed at least twice to ensure experiment reproducibility.

**TABLE 1:** **Oligonucleotides used to study *Thermococcus* sp. 9°N polD kinetics** Boldface letters indicate base opposite incoming nucleotide. Underlined letters indicate change from Template-1 to Template-2.

Name	Sequence
5′-FAM primer	5′-FAM-AGT GAA TTC GAG CTC GGT ACC CGG GGA TCC TCT AGA GTC GAC CTG CAG GT-3′
G Template-1	5′-CCC TAA TCA TAT CCT A**G**A CCT GCA GGT CGA CTC TAG AGG ATC CCC GGG TAC CGA GCT CGA ATT CAC T-3′
A Template-1	5′-TTG CTC GTT TGC TGG G**A**A CCT GCA GGT CGA CTC TAG AGG ATC CCC GGG TAC CGA GCT CGA ATT CAC T-3′
T Template-1	5′-AAG CAC GAA AGC AGG G**T**A CCT GCA GGT CGA CTC TAG AGG ATC CCC GGG TAC CGA GCT CGA ATT CAC T-3′
C Template-1	5′-AAG TAT GAA AGT AGG G**C**A CCT GCA GGT CGA CTC TAG AGG ATC CCC GGG TAC CGA GCT CGA ATT CAC T-3′
G Template-2	5′-CCC TAA TCA TAT CCT T**G**A CCT GCA GGT CGA CTC TAG AGG ATC CCC GGG TAC CGA GCT CGA ATT CAC T-3′
A Template-2	5′-TTG CTC GTT TGC TGG C**A**A CCT GCA GGT CGA CTC TAG AGG ATC CCC GGG TAC CGA GCT CGA ATT CAC T-3′
T Template-2	5′-AAG CAC GAA AGC AGG C**T**A CCT GCA GGT CGA CTC TAG AGG ATC CCC GGG TAC CGA GCT CGA ATT CAC T-3′
C Template-2	5′-AAG TAT GAA AGT AGG A**C**A CCT GCA GGT CGA CTC TAG AGG ATC CCC GGG TAC CGA GCT CGA ATT CAC T-3′

##### polD^−^ Pre-steady-state Single Nucleotide Incorporation

To obtain the rates of correct nucleotide incorporation, incorrect nucleotide incorporation, and ribonucleotide incorporation by polD^−^, pre-steady-state single nucleotide assays were performed. The primer·template substrates used in these assays were prepared as described above. A 150-μl polD^−^/DNA aliquot was prepared by mixing ThermoPol buffer (1× final concentration), primer·template DNA (30 nm final concentration), and a 3-fold excess of polD^−^ (90 nm active enzyme final concentration); a control experiment demonstrated that polymerase saturation was reached at 3-fold excess polD^−^, satisfying pre-steady-state requirements (data not shown). A second 150-μl aliquot was prepared by mixing ThermoPol buffer (1× final concentration) and dNTP (5–200 μm). Higher concentrations (100–4000 μm) were required for incorrect nucleotide and ribonucleotide incorporation assays. Using the RQF, the polD^−^/DNA construct was rapidly mixed with dNTP and quenched with 50 mm EDTA. After mixing an equal volume of polD^−^/DNA and dNTP solutions by the RQF, the final reaction concentrations were 15 nm DNA, 45 nm active polD^−^, and 2.5–100 μm dNTP for correct nucleotides (or 50–2000 μm dNTP for incorrect nucleotides or ribonucleotides) in 1× ThermoPol buffer. A control reaction was performed in which dNTP was replaced with 1× ThermoPol buffer and reacted in the RQF with polD^−^/DNA for 10 s. Reaction products were separated by capillary electrophoresis as described above.

Because reaction products for dCTP incorporation did not resolve into two fully distinguishable substrate and product peaks by capillary electrophoresis, we instead separated the products of dCTP insertion by denaturing gel electrophoresis (20% polyacrylamide, 8 m urea, 1× TBE buffer), visualized by Typhoon TRIO (GE Healthcare), and quantitated with ImageQuant software (Molecular Dynamics). In order to confirm that both capillary and gel electrophoresis give comparable results, the 100 μm dTTP incorporation time course was analyzed by both capillary electrophoresis and gel electrophoresis (data not shown).

The product concentration was graphed as a function of time, and the data were fit to the single-exponential Equation 2 to obtain the observed rate constant of nucleotide incorporation (*k*_obs_) using the nonlinear regression program Kaleidagraph (Synergy Software).

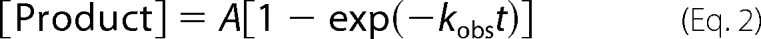
 To obtain the maximum rate of polymerization constant (*k*_pol_)and apparent equilibrium dissociation constant (*K_d_*_(dNTP)_), the *k*_obs_ values were graphed as a function of dNTP (or rNTP) concentration, and the data were fit to the hyperbolic Equation 3 using Kaleidagraph.

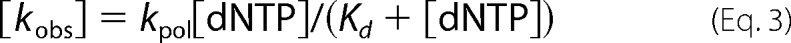
 The specific activities for correct nucleotide, incorrect nucleotide, and ribonucleotide incorporation were calculated using Equation 4, and the nucleotide selectivities for incorrect nucleotide incorporation and ribonucleotide incorporation were calculated using Equation 5.

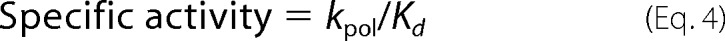


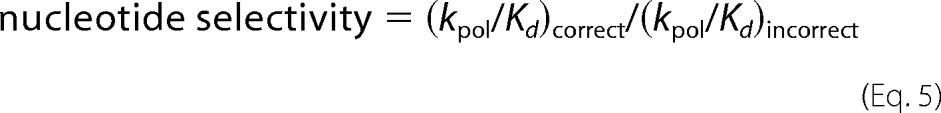


##### polD^−^ Pyrophosphorolysis

Under certain circumstances, DNA polymerases can perform the reverse reaction (pyrophosphorolysis) to remove nucleotides from the 3′-end of the DNA molecule in the absence of exonuclease activity. To obtain the rate of polD^−^ pyrophosphorolysis, a 3-fold excess of polD^−^ was preincubated with 5′-FAM primer·C Template-1 and was rapidly mixed with 50–500 μm (final concentration after RQF mixing) inorganic pyrophosphate (PP_i_) using the RQF at 60 °C. Reaction products were separated by capillary electrophoresis and analyzed as described above. The concentration of pyrophosphorolysis product (<50 nt of DNA) was graphed as a function of time and fit to Equation 2 to obtain the observed rate constants (*k*_obs_), followed by fitting the *k*_obs_ as function of PP_i_ concentration in Equation 3 to obtain a maximal rate for pyrophosphorolysis (*k*_pyro_) and apparent equilibrium dissociation constant of PP_i_ (*K_d_*_(PPi)_). In reactions with multiple pyrophosphorolysis events, all product peaks were summed as total product.

##### polD 3′-5′ Exonuclease Activity

The kinetics of polD 3′-5′ exonuclease activity was measured by monitoring shortening of a FAM-labeled DNA primer. To obtain the rate of 3′-5′ exonuclease activity on single-stranded DNA, a pre-steady-state single-turnover assay was performed. A 40 nm aliquot of 5′-FAM primer was prepared in 1× ThermoPol II buffer (20 mm Tris-HCl, 10 mm (NH_4_)_2_SO_4_, 10 mm KCl, 0.1% Triton X-100, pH 8.8, at 25 °C) supplemented with either 2 mm MgSO_4_ or MnSO_4_. A second aliquot containing polD (120 nm) was prepared in 1× ThermoPol II buffer supplemented with either 2 mm MgSO_4_ or MnSO_4_. Using the RQF, polD and 5′-FAM labeled primer were rapidly mixed at 60 °C from 0.002 to 5 s and quenched with 0.1 n H_2_SO_4_. Final reaction conditions after mixing by the RQF were 20 nm primer, 60 nm polD in 1× ThermoPol II buffer with 2 mm MgSO_4_ or MnSO_4_. After quenching, samples were neutralized with 1 n NH_4_OH, and reaction products were separated by capillary electrophoresis and analyzed by peak scanner as described above. All product peaks (<50 nt) resulting from 3′-5′ exonuclease hydrolysis were summed and graphed as a function of time, followed by fitting of data to Equation 2 as described previously. It is important to note that 0.05, 0.1, and 1 mEDTA did not sufficiently quench 3′-5′ exonuclease reactions at rapid time scales, but 0.1 n H_2_SO_4_ was sufficient to quickly and reliably quench activity (data not shown).

## Results

### 

#### 

##### Analysis of polD^−^ Multiple-turnover Steady-state Nucleotide Incorporation Kinetics Reveals a Postchemistry Rate-limiting Step

**FIGURE 1.**
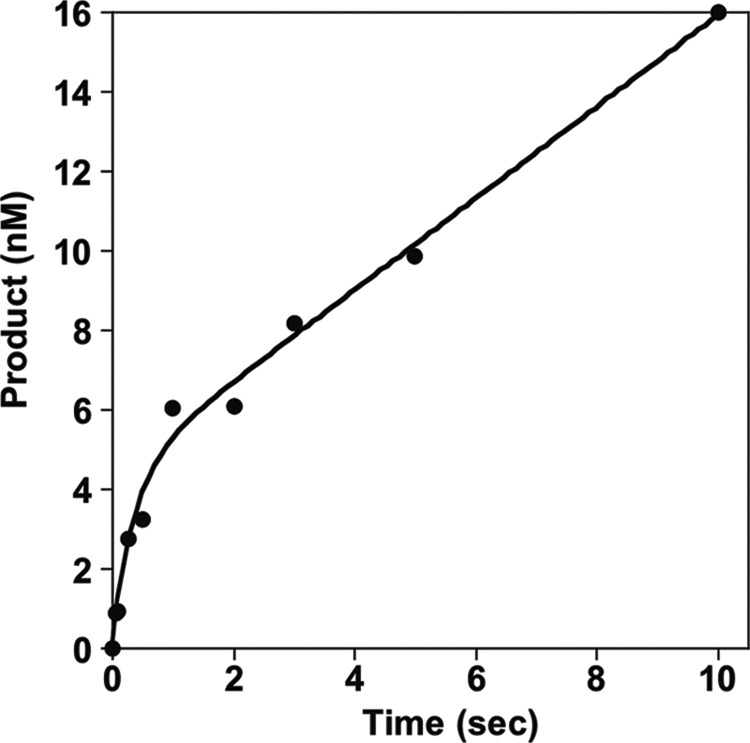
**polD^−^ steady-state dGTP incorporation.** A 4-fold excess of 50 nt 5′-FAM primer·C Template-1 was preincubated with polD^−^ and rapidly mixed with dGTP followed by quenching with 50 mm EDTA using an RQF at 60 °C. The yield of 51-nt product was graphed as a function of time and fit to Equation 1 to obtain *k*_ss_ = *k*_2_/*A* of 0.3 s^−1^, a *k*_obs_ of 2.8 s^−1^, and an active enzyme concentration (*A*) of 4.8 nm.

Steady-state kinetic assays are used to infer the rate-determining step of a catalytic pathway and are performed under multiple-turnover conditions (*i.e.* [substrate] ⋙ [enzyme]). Previous steady-state kinetic studies performed on a host of different DNA polymerases have revealed that nucleotide incorporation is fast and DNA release is rate-limiting (21–27). In order to determine if the same is true for polD^−^, a steady-state multiple-turnover assay was performed in which polD^−^ was preincubated with a 4-fold excess of DNA and rapidly mixed with dGTP at 60 °C using an RQF. Under these conditions, only one dGTP is added to the primer because the next position requires incorporation of a nucleotide that is absent in the reaction mixture. polD^−^ must then dissociate from the DNA and bind to a new primer·template to repeat the addition of the single dGTP. DNA substrate and product were analyzed by capillary electrophoresis. Concentration of the 51-nt DNA product was graphed as a function of time, and the steady-state kinetic parameter, *k*_ss_, was determined. We observe a rapid initial accumulation of product, designated the burst phase, followed by a slower linear phase of product formation (Fig. 1). The burst phase represents the fast chemistry step of nucleotide incorporation and is proportional to the concentration of the active prebound polymerase. The linear phase represents a slow step occurring after chemistry. This slow phase, probably dominated by the rate of DNA release between nucleotide addition reactions, defines the polD^−^ steady-state turnover rate, 0.3 s^−1^ (Table 2). Furthermore, by extrapolating the linear phase through the *y* axis, the concentration of active enzyme can be determined. For this preparation of polD^−^, 4.8 nm of the 10 nm was active. This active enzyme concentration (4.8 nm) was used to calculate the active polD^−^ concentration in subsequent pre-steady-state studies using this preparation of enzyme.

**TABLE 2:** **Steady-state and pre-steady-state kinetic parameters of *Thermococcus* sp. 9°N polD** Polymerization *k*_pol_ and *K_d_*^(dNTP)^ values represent the ranges for all four dNTPs. Error is derived from the fit to the corresponding equation.

Reaction observed	Parameter	Value
DNA release	*k*_ss_	0.30 ± 0.01 s^−1^
Polymerization	*k*_pol_	1.8–3.1 s^−1^
	*K_d_*_(dNTP)_	0.9–2.5 μm
Pyrophosphorolysis	*k*_pyro_	0.40 ± 0.03 s^−1^
	*K_d_*_(PPi)_	190 ± 20 μm
ssDNA 3′-5′ exonuclease	*k*_exo_^Mg2+^	≥2.5 ± 0.3 s^−1^
	*k*_exo_^Mn2+^	≥110 ± 10 s^−1^

Several factors may account for the discrepancy between total and active enzyme concentrations. The concentration of polD^−^ was first determined spectrophotometrically, which may account for inaccurate apparent protein concentration. Alternatively, incorrect stoichiometry between small and large subunits, misfolding of the subunits, or multiple DNA polymerases binding to a single DNA substrate may also account for a low apparent active enzyme concentration.

##### Analysis of polD^−^ Pre-steady-state Single-turnover Correct Nucleotide Incorporation Kinetics Reveals a Slow Rate of Incorporation and Tight Nucleotide Binding

**FIGURE 2.**
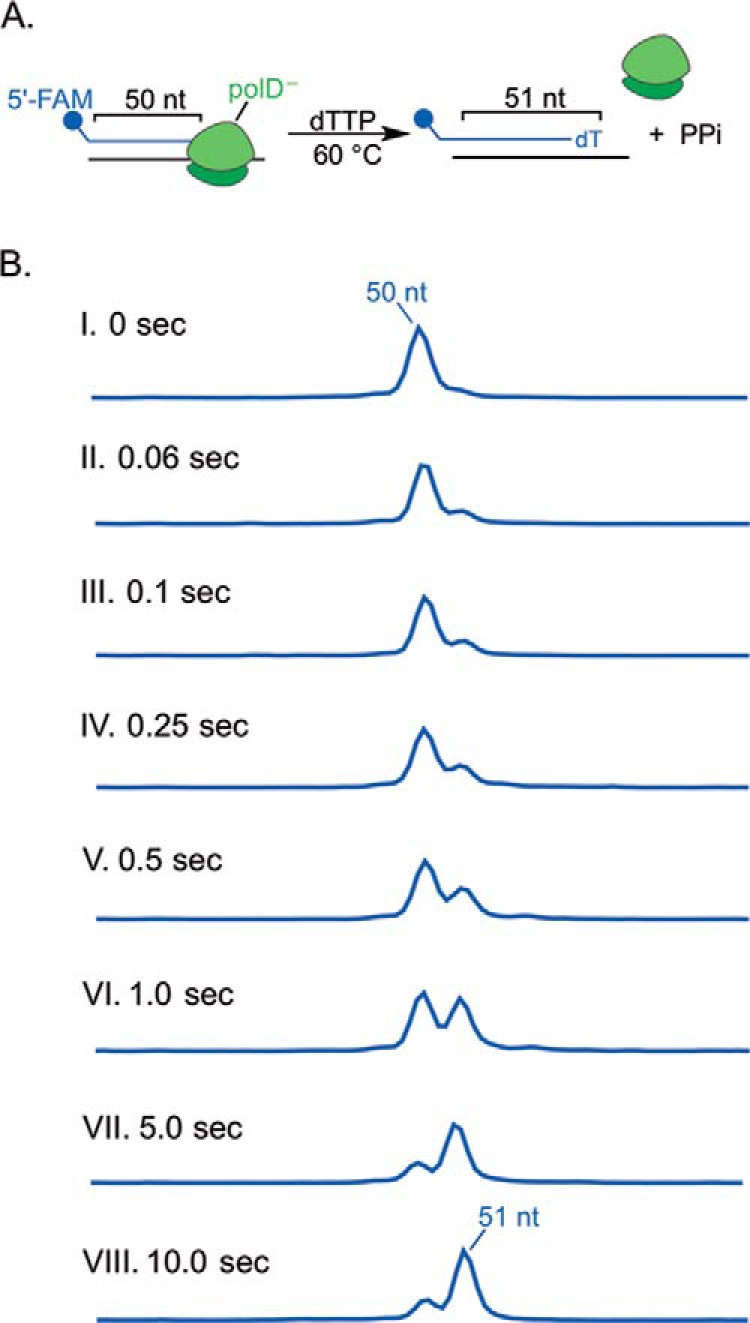
**polD^−^ pre-steady-state dTTP incorporation.** *A*, *reaction scheme*, a 50-nt 5′-FAM primer was annealed to template DNA and then preincubated with a 3-fold excess of active polD^−^, followed by rapid mixing with dTTPs at 60 °C and incubation from 0 to 10 s. Because only dTTP is present, the primer can only be extended 1 nt unless an incorrect nt is added. *B*, substrates (50 nt) and incorporation products (51 nt) were resolved by capillary electrophoresis after denaturation of the double-stranded DNA.

Pre-steady-state kinetic assays are performed under single-turnover conditions (*i.e.* [enzyme] ⋙ [substrate]) and are used to determine the kinetic parameters associated with steps masked by the rate-limiting step, such as single nucleotide incorporation (7). To obtain rates for nucleotide incorporation, including maximal rate of polymerization, *k*_pol_, and apparent equilibrium dissociation constant, *K_d_*_(dNTP)_, polD^−^ pre-steady-state kinetic assays were performed. Such parameters provide insight into how polymerases discriminate against incorrect nucleotides and incorporate correct nucleotides. The *k*_pol_ reflects how fast the polymerase will incorporate a nucleotide, whereas the *K_d_*_(dNTP)_ reflects how tightly the polymerase binds a nucleotide, where a lower *K_d_* reflects tighter nucleotide binding. Although *k*_pol_ and *K_d_*_(dNTP)_ can be obtained from the burst phase of steady-state kinetics, such methods may result in parameters with large sources of error (5). Therefore, to measure pre-steady-state kinetics, a 3-fold excess of active polD^−^ was preincubated with DNA, rapidly mixed with single dNTP solutions at 60 °C, and analyzed as described above. A schematic of the incorporation assay and expected capillary electrophoresis results are depicted in Fig. 2. For each dNTP concentration, the concentration of product was graphed as a function of time to obtain the observed rate of nucleotide incorporation, *k*_obs_. The *k*_pol_ as well as the *K_d_*_(dNTP)_ for each dNTP was obtained by graphing *k*_obs_ *versus* dNTP concentration.

**FIGURE 3.**
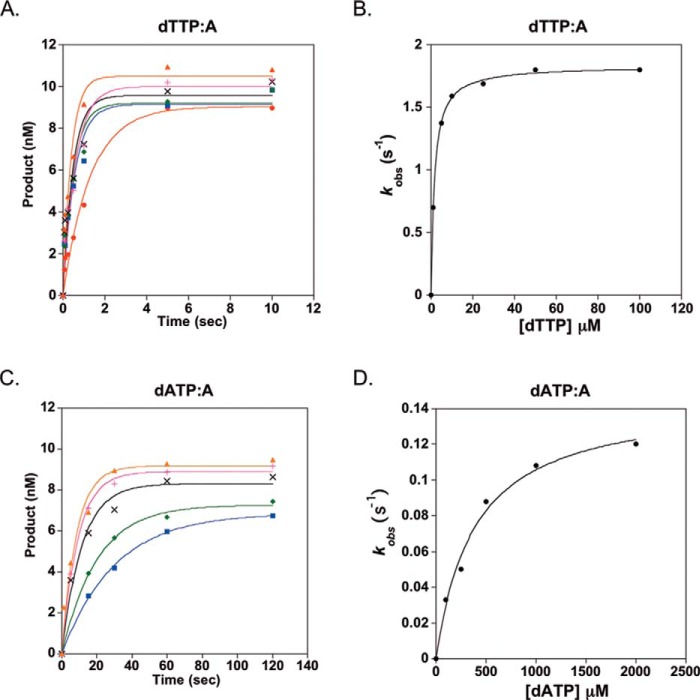
**polD^−^ pre-steady-state kinetics of correct and incorrect nucleotide incorporation.** *A*, a 50-nt 5′-FAM primer was annealed to A Template-1 DNA and preincubated with a 3-fold excess of active polD^−^, followed by rapid mixing at 60 °C with 2.5 (●), 5 (■), 10 (♦), 25 (×), 50 (+), or 100 (▴) μm (final concentration) dTTP, creating correct T:A base pairing. The 51-nt product was graphed as a function of time and fit to Equation 2 to obtain *k*_obs_. *B* and *D*, the dependence of reaction rate *k*_obs_ on nucleotide concentration was fit to a hyperbolic equation (Equation 3) to obtain *k*_pol_ and *K_d_*_(dNTP)_ for correct dTTP:A (*B*) and incorrect dATP:A incorporation (*D*). *C*, primer·template DNA was prepared and preincubated with polD^−^ as described above and mixed with 100 (■), 250 (♦), 500 (×), 1000 (+), or 2000 (▴) μm (final concentration) dATP, creating incorrect A:A base pairing, and fit to the above Equation 2 to obtain *k*_obs_.

As shown in Fig. 3*A* for dTTP incorporation paired with template dA (dTTP:A), we observe an increase in *k*_obs_ with increasing dTTP concentration, where we reach a maximal *k*_obs_ at high (100 μm) dTTP concentration. When *k*_obs_ is graphed as a function of dTTP concentration, the maximal rate of polymerization is obtained from the plateau, whereas the *K_d_*_(dNTP)_ is the dTTP concentration at half *k*_pol_ (Fig. 3*B*).

Table 3 shows the obtained *k*_pol_ and *K_d_*_(dNTP)_ values for correct nucleotide incorporation using separate matched templates for each possible dNTP. All values for *k*_pol_ are within 2-fold, ranging from 1.8 to 3.1 s^−1^, suggesting that polD incorporates all correctly base-paired nucleotides at a similar rate. Similarly, the obtained *K_d_*_(dNTP)_ values are within 3-fold, ranging from 0.9 to 2.5 μm, indicating that polD binds each correct nucleotide with similar affinity (Table 3). Furthermore, we calculated the specificity constant for each correct nucleotide, obtained by dividing *k*_pol_ by the corresponding *K_d_*_(dNTP)_ (Table 3). The specificity constant (*k*_pol_/*K_d_*_(dNTP)_), a reflection of both the incorporation rate and dNTP binding, is used to compare the efficiency of incorporation for each nucleotide by a polymerase as well as to compare the efficiency of incorporation with other polymerases. A larger specificity constant reflects more efficient binding and incorporation (5). For polD^−^, specificity constants for correct nucleotide incorporation range from 1.0 to 2.3 μm^−1^ s^−1^ (Table 3), again demonstrating that the enzyme lacks preference among Watson-Crick base-paired dNTP substrates.

**TABLE 3:** **Pre-steady-state single nucleotide kinetic parameters of *Thermococcus* sp. 9°N polD** Correctly incorporated nucleotides are shown in boldface type. Independent experiments were performed at least twice to ensure reproducibility. Reported values are from a typical experiment, and error is derived from fit to the hyperbolic equation.

dNTP	*k*_pol_	*K_d_*	*k*_pol_/*K_d_*	Nucleotide selectivity*^a^*
	*s*^−*1*^	μ*m*	μ*m*^−*1*^*s*^−*1*^	
**Template A**				
**dTTP**	**1.8 ± 0.1**	**1.6 ± 0.1**	**1.1**	
dATP	0.15 ± 0.01	390 ± 70	3.9 × 10^−4^	2.8 × 10^3^
dCTP	0.22 ± 0.02	450 ± 30	4.9 × 10^−4^	2.2 × 10^3^
dGTP	0.18 ± 0.01	330 ± 80	5.5 × 10^−4^	2.0 × 10^3^

**Template G**				
**dCTP**	**3.1 ± 0.8**	**1.7 ± 0.1**	**1.8**	
dATP	0.12 ± 0.01	320 ± 70	3.8 × 10^−4^	4.7 × 10^3^
dTTP	0.38 ± 0.04	650 ± 150	5.9 ×10^−4^	3.0 × 10^3^
dGTP	0.07 ± 0.01	320 ± 80	2.2 × 10^−4^	8.1 × 10^3^

**Template C**				
**dGTP**	**2.1 ± 0.1**	**0.9 ± 0.3**	**2.3**	
dATP	1.3 ± 0.1	570 ± 140	2.3 × 10^−3^	1.0 × 10^3^
dTTP	0.55 ± 0.09	1400 ± 500	3.9 × 10^−4^	5.9 × 10^3^
dCTP	0.42 ± 0.02	390 ± 120	1.0 × 10^−3^	2.3 × 10^3^

**Template T**				
**dATP**	**2.6 ± 0.7**	**2.5 ± 0.1**	**1.0**	
dTTP	0.28 ± 0.02	490 ± 80	5.7 × 10^−4^	1.8 × 10^3^
dCTP	0.35 ± 0.03	530 ± 90	6.6 × 10^−5^	1.5 × 10^4^
dGTP	0.11 ± 0.01	300 ± 60	3.6 × 10^−4^	2.8 × 10^3^

*^a^* Calculated as (*k*_pol_/*K_d_*)_correct_/(*k*_pol_/*K_d_*)_incorrect_.

##### Pre-steady-state Kinetic Analysis of polD^−^ Incorrect Nucleotide Incorporation Reveals the Presence of a Nucleotide Discrimination Mechanism

DNA polymerases have evolved specific mechanisms to discriminate against the incorporation of incorrect nucleotides during synthesis (reviewed in Ref. 4). Such mechanisms are critical for faithful replication of the genome and to ensure the transfer of accurate information to subsequent generations. Importantly, nucleotide selectivity mechanisms have not been characterized in polD. Therefore, we performed a thorough kinetic characterization of incorrect nucleotide incorporation to understand polD fidelity. Pre-steady-state single-turnover kinetic experiments were performed on the 12 possible incorrect base pairing combinations (Table 3).

As shown in Fig. 3*C* for dATP:A incorporation for dATP incorporation paired with template dA, *k*_obs_ increases with higher dATP concentrations. It is important to note that maximal *k*_obs_ for dATP:A incorporation is reached at exceedingly high dATP concentration, 2000 μm, compared with 100 μm for correct dTTP:A incorporation. Furthermore, maximal product incorporation is achieved after 60 s for incorrect dATP:A incorporation, 12 times slower than for correct dTTP:A incorporation. By plotting the observed *k*_obs_ *versus* dATP concentration, the *k*_pol_ and *K_d_*_(dNTP)_ for incorrect nucleotide incorporation were obtained (Fig. 3*D* and Table 3). These results suggest that for misincorporation, high substrate concentrations and longer reaction times are required to drive the incorporation of the incorrect nucleotide and imply that polD contains specific mechanisms to exclude incorrect nucleotides, similar to other DNA polymerases.

Table 3 shows *k*_pol_ and *K_d_*_(dNTP)_ values for all 12 incorrect nucleotide incorporation combinations. Obtained *k*_pol_ rates range from 0.07 to 1.3 s^−1^, resulting in a 1.5–45-fold decrease in *k*_pol_ between correct and incorrect dNTP incorporation (Table 3). The *K_d_*_(dNTP)_ ranges from 300 to 1400 μm, a 120–1500-fold increase in *K_d_*_(dNTP)_ between correct and incorrect binding (Table 3). These results suggest that polD^−^ prevents the incorporation of incorrect nucleotides primarily through weaker binding of the incorrect nucleotide, as reflected in the large increase in *K_d_*.

As done for correct nucleotide incorporation assays, the specificity constant was calculated for incorrect nucleotide incorporation. Nucleotide selectivity, obtained by dividing the specificity constant for the correct nucleotide by the specificity constant for the incorrect nucleotide, reflects overall DNA polymerase fidelity (5). Of the 12 potential mismatches, polD discriminates most strongly against a dGTP:G mismatch (8100-fold) and weakly discriminates against a dATP:C mismatch (1000-fold) (Table 3).

##### Analysis of polD^−^ Single-turnover Ribonucleotide Incorporation Kinetics Reveals the Presence of a Ribonucleotide Discrimination Mechanism

In addition to incorrect nucleotide discrimination, polymerases discriminate against ribonucleotide incorporation (28, 29). Excluding rNTPs is especially important due to the intracellular excess of rNTPs over dNTPs (30). Furthermore, rNTPs incorporated during replication may lead to genome instability by inducing strand breaks. Therefore, to understand whether and how polD^−^ discriminates against ribonucleotides, a pre-steady-state single-turnover assay was performed examining rATP incorporation paired to dT (rATP:T) as described above. For rATP incorporation, *k*_pol_ was 0.16 s^−1^ and *K_d_*_(rNTP)_ was 360 μm, which correspond to a 16-fold decrease in *k*_pol_ and a 144-fold increase in *K_d_*_(rNTP)_ compared with incorporation of dATP (Table 4). Such results indicate that ribonucleotide discrimination occurs primarily through reduced binding affinity of the rNTP. The specificity constant (*k*_pol_/*K_d_*_(rNTP)_), 4.4 × 10^−4^, and nucleotide selectivity constant ((*k*_pol_/*K_d_*_(dNTP)_)/(*k*_pol_/*K_d_*_(rNTP)_)), 2.3 × 10^3^, suggest that dATP is incorporated 2,300-fold more efficiently than rATP (Table 4).

**TABLE 4:** **Comparison of ribonucleotide incorporation kinetics for polymerase families A, B, D, Y, and RT**

Polymerase	Family	*k*_pol_	*K_d_*_(rNTP)_	*k*_pol_/*K_d_*_(rNTP)_	Selectivity
		*s*^−*1*^	μ*m*	μ*m*^−*1*^*s*^−*1*^	
KF (45)	A	(4.7 ± 2.5) × 10^−2^	21 ± 7	2.3 × 10^−3^	3.4 × 10^3^
RB69 (24)	B	0.74 ± 0.2	(1.6 ± 0.4) × 10^−4^	4.6 × 10^−5^	6.4 × 10^4^
9°N polD (this work)	D	0.16 ± .01	360 ± 60	4.4 × 10^−4^	2.3 × 10^3^
Dbh (47)	Y	(1.4 ± 0.3) × 10^−5^	770 ± 360	1.8 × 10^−8^	3.4 × 10^3^
HIV-1 (49)	RT	0.03	820 ± 150	3.7 × 10^−5^	1.3 × 10^5^

##### Analysis of polD^−^ Pyrophosphorolysis

**FIGURE 4.**
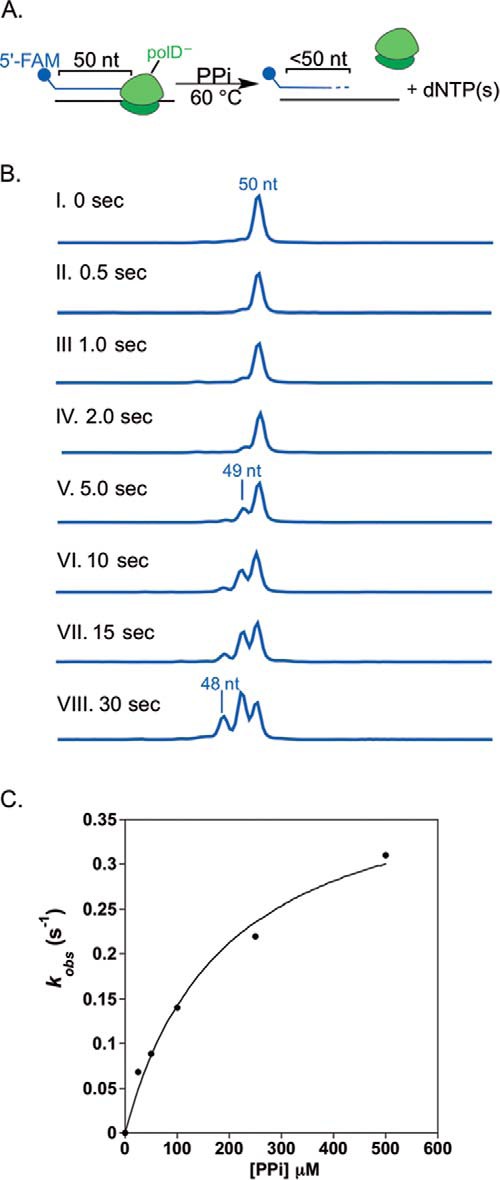
**polD^−^ pyrophosphorolysis.** *A*, *reaction scheme*, a 50-nt 5′-FAM primer was annealed to template DNA and preincubated with a 3-fold excess of active polD^−^ followed by rapid mixing with PP_i_ at 60 °C using an RQF. Pyrophosphorolysis results in production of a smaller FAM-labeled oligonucleotide. *B*, pyrophosphorolysis products (<50 nt) were resolved by capillary electrophoresis after DNA denaturation. *C*, the dependence of the reaction rate *k*_obs_ on PP_i_ concentration was fit to Equation 3 to obtain the maximum rate of *k*_pyro_ and the equilibrium binding constant of *K_d_*_(PPi)_, ∼0.4 s^−1^ and ∼190 μm, respectively.

During the incorporation of dNTPs by a DNA polymerase, PP_i_ is produced and released. If PP_i_ stays bound within the enzyme active site, reversal of chemistry can occur, resulting in pyrophosphorolysis, which shortens the primer and releases a dNTP. In the presence of a high concentration of PP_i_, the polymerase can remove multiple dNTPs during a single DNA polymerase-DNA binding event. Single-turnover kinetic assays were performed to test whether polD^−^ undergoes pyrophosphorolysis and to obtain the maximal rate of pyrophosphorolysis, *k*_pyro_, and the apparent dissociation rate constant for PP_i_, *K_d_*_(PPi)_. A schematic of the pyrophosphorolysis assay and the expected capillary electrophoresis results are shown in Fig. 4. We observed pyrophosphorolysis of multiple nucleotides at longer time points (Fig. 4*B*, *VI–VIII*). Concentration of product (<50 nt of DNA) was graphed as a function of time to obtain the observed rate constants, *k*_obs_ (data not shown). To obtain *k*_pyro_ and *K_d_*_(PPi)_, *k*_obs_ was graphed as a function of PP_i_ concentration (Fig. 4*C*). For polD^−^, *k*_pyro_ is 0.4 s^−1^ and *K_d_*_(PPi)_ is 190 μm (Table 2).

##### Analysis of polD 3′-5′ Exonuclease Kinetics Reveals a 40-Fold Preference for Mn^2+^ over Mg^2+^

**FIGURE 5.**
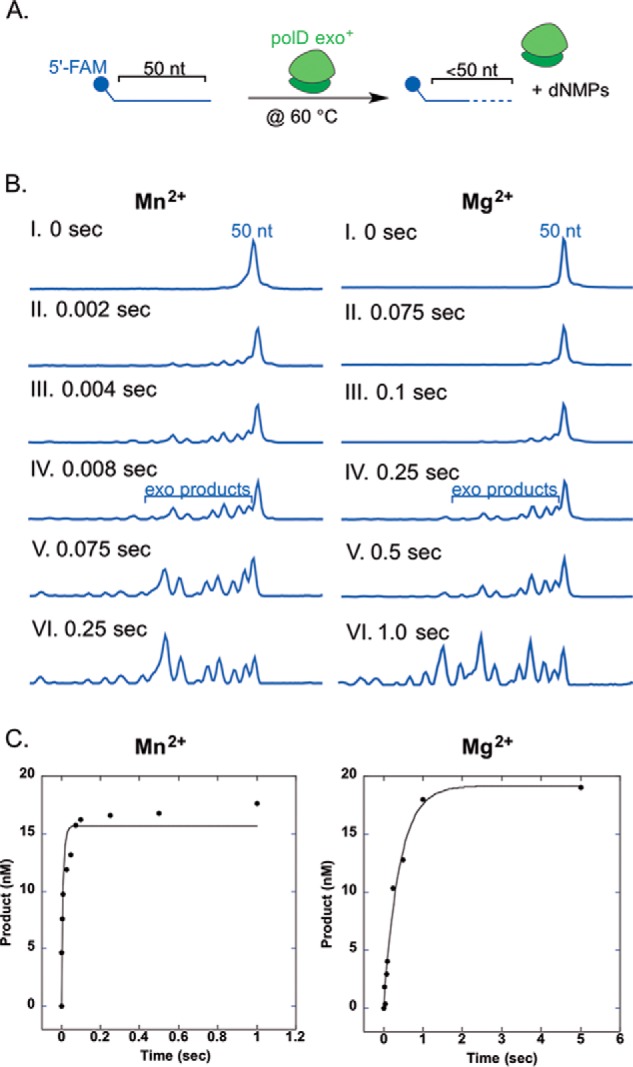
**polD 3′-5′ exonuclease kinetics.** *A*, *reaction scheme*, a 50-nt single-stranded 5′-FAM primer was rapidly mixed with a 3-fold excess of polD in the presence of either MnSO_4_ or MgSO_4_ at 60 °C and quenched with 0.1 n H_2_SO_4_ using an RQF. *B*, expected capillary electrophoresis results for processive polD exonuclease hydrolysis in the presence of MnSO_4_ or MgSO_4_. *C*, product, <50 nt DNA, was graphed as a function of time and fit to Equation 2 to obtain the rate of polD exonuclease hydrolysis, *k*_exo_, in the presence of MnSO_4_ or MgSO_4_, ∼110 and 2.5 s^−1^, respectively.

Along with nucleotide incorporation activity, many polymerases, including polD, contain a metal-dependent 3′-5′ exonuclease activity, which facilitates removal of incorrectly incorporated nucleotides, thereby increasing the overall fidelity of the polymerase (31). *In vivo*, a DNA polymerase melts duplex DNA to shuttle the single-stranded DNA primer to the exonuclease active site prior to hydrolysis (31). Importantly, melting of duplex DNA during DNA polymerase exonuclease hydrolysis was previously determined to be rate-limiting (32). In order to focus on 3′-5′ exonuclease hydrolysis rates rather than DNA duplex melting dynamics, experiments were designed using a single-stranded DNA substrate (32). To assess polD exonuclease activity in the presence of Mn^2+^ and Mg^2+^, we performed pre-steady-state assays in which we rapidly mixed a 3-fold excess of polD with single-stranded 5′-FAM DNA in the presence of either metal ion. A schematic of this assay is shown in Fig. 5*A*, and representative CE traces for exonuclease kinetics done in the presence of Mn^2+^ or Mg^2+^ are shown in Fig. 5*B*. The concentration of exonuclease product (<50 nt of DNA) was graphed as a function of time and fit to a single-exponential burst equation to obtain pre-steady-state kinetic rates of exonuclease hydrolysis (Fig. 5). Due to rapid exonuclease hydrolysis by polD, multiple dNMPs were excised from the 5′-FAM substrate at each reaction time. We therefore report a lower limit for polD *k_exo_*, ≥110 and ≥2.5 s^−1^, in the presence of Mn^2+^ and Mg^2+^, respectively. The results obtained here suggest that there is a ≥44-fold increase in the exonuclease hydrolysis rate in the presence of Mn^2+^ compared with Mg^2+^ (Table 2). Several alternative RQF experiments were unsuccessful in limiting polD exonuclease hydrolysis to removal of a single dNMP, including prebinding polD to 5′-FAM ssDNA and reaction initiation by the addition of Mn^2+^ or Mg^2+^ in the presence of a large excess of trap DNA to prevent rebinding to the FAM-labeled DNA (data not shown).

## Discussion

### 

#### 

##### Family D DNA Polymerase Kinetic Scheme

**FIGURE 6.**
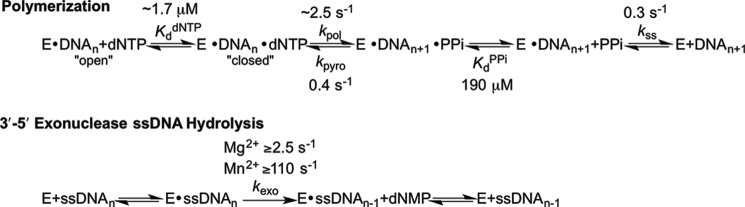
**Schematic of polD kinetic pathway.** During polymerization (top pathway), DNA polymerase binds to DNA (*E*·DNA*_n_*), followed by dNTP binding (*E*·DNA*_n_*·dNTP). A proposed conformational change from an “open” polymerase conformation to a “closed” conformation brings the α-phosphate of the bound dNTP within close proximity to the 3′-OH of the primer DNA. Here, nucleotide incorporation chemistry occurs when the 3′-OH of primer DNA attacks the α-phosphate of the bound dNTP, incorporating dNMP, extending the primer DNA by 1 nucleotide and generating PP_i_ (*E*·DNA*_n_*_+1_·PP_i_). Following nucleotide incorporation, the polymerase undergoes two proposed conformation changes to release PP_i_ and then DNA (*E* + DNA*_n_*_+1_). Alternatively, during 3′-5′ exonuclease hydrolysis (*bottom pathway*), the 3′-5′ exonuclease active site binds ssDNA (ssDNA*_n_*) and hydrolysis occurs, shortening the DNA to release a dNMP from the enzyme·DNA complex (*E*·ssDNA*_n_*_− 1_). Kinetic constants derived from this study are averages from Table 3.

The overall polymerization kinetic pathway is highly conserved among different DNA polymerase families (Fig. 6, *top path*). In this pathway, DNA polymerase first binds to DNA (*E*·DNA*_n_*), followed by dNTP binding (*E*·DNA*_n_*·dNTP), which induces a conformational change from an “open” polymerase conformation to a “closed” conformation. Once in a closed conformation, the α-phosphate of the bound dNTP is within close proximity to the 3′-OH of primer DNA. Here, nucleotide incorporation chemistry occurs when the 3′-OH of primer DNA attacks the α-phosphate of bound dNTP, incorporating dNMP and generating PP_i_ (*E*·DNA*_n_*_+ 1_·PP_i_). Following nucleotide incorporation, the polymerase undergoes two conformation changes to release PP_i_ and then DNA (*E* + DNA*_n_*_+ 1_). All steps within the polymerase kinetic scheme are reversible. Furthermore, it is generally agreed upon that the conformational changes are faster than chemistry, and DNA release is the slow rate-limiting step of the kinetic pathway (5).

polD, however, is structurally and genetically unrelated to any other DNA polymerase family. Notably, there are few structural or functional studies for this family of polymerases in the literature to date. Thus, caution must be applied in making assumptions about the polD reaction mechanism through analogy to other studied polymerases. To address this issue, we have performed a variety of kinetic assays to elucidate the polD reaction pathway and allow comparisons with other polymerase families. Our findings demonstrate that polD does, in fact, follow a similar overall polymerization kinetic pathway as other families of DNA polymerases.

Under multiple-turnover conditions, we observed a burst kinetic profile, indicating that polD shares the feature of fast nucleotide incorporation chemistry followed by a rate-limiting post-phosphoryl transfer reaction. Pre-steady-state kinetic experiments revealed that the *k*_pol_ is 1.8–3.1 s^−1^ and the *K_d_*_(dNTP)_ is 0.9–2.5 μm (Table 2) for all correct nucleotide base-pairing combinations. Furthermore, pre-steady-state pyrophosphorolysis kinetics revealed that nucleotide incorporation is indeed reversible, providing further evidence that Family D DNA polymerases follow the generally observed cross-family polymerase kinetic scheme (Table 2 and Fig. 6). Importantly, maximal nucleotide incorporation rates are faster than maximal pyrophosphorolysis rates, and the *K_d_*_(dNTP)_ for correct nucleotide binding is 95-fold tighter than *K_d_*_(PPi)_, suggesting that the equilibrium of correct nucleotide incorporation chemistry strongly favors dNTP incorporation over dNTP removal (Table 2).

Although polD has been implicated as a replicative polymerase in Euryarchaea, the maximal rate of polymerization, *k*_pol_, for polD is one of the slowest for kinetically characterized replicative polymerases (Family A, B, and C) and repair and translesion polymerases (Family X and Y) (Table 5). On the contrary, polD has one of the smallest, and therefore tightest, *K_d_*_(dNTP)_ values of kinetically characterized polymerases (Table 5). Importantly, the resulting specific activity for polD, ∼1.8 μm^−1^ s^−1^, is average compared with other replicative, repair, and translesion polymerases (Table 5).

**TABLE 5:** **Comparison of pre-steady-state nucleotide incorporation kinetics for polymerase families A, B, C, D, X, Y, and RT**

Polymerase	*k*_pol_	*K_d_*_(dNTP)_	*k*_pol_/*K_d_*
	*s*^−*1*^	μ*m*	μ*m*^−*1*^ *s*^−*1*^

**Family A**			
KF (21)	50	5.5	9.0
T7 (50)	120	2	60
Klentaq (51)	21	35	0.60

**Family B**			
Vent polB (25)	66	70	0.95
RB69 (52)	200	69	2.9
T4 (53)	>400	20	20
Human pol ϵ (27)	248	31	8
Yeast pol δ (54)	0.93	24	0.04

**Family C**			
Sau PolC (55)	180	4	45

**Family D**			
9°N polD (this work)	3.1	1.7	1.8

**Family X**			
rPolβ (23)	12.5	1.9	6.6

**Family Y**			
Dpo4 (40)	7.6	70	0.10

**Reverse transcriptase**			
HIV-1 (56)	26	9	2.88

##### Metal Ion Dependence of polD 3′-5′ Exonuclease ssDNA Hydrolysis

The proposed 3′-5′ exonuclease ssDNA hydrolysis kinetic pathway for polD is presented in Fig. 6 (*bottom path*). Importantly, polD exonuclease hydrolysis requires a divalent metal ion for catalysis, typically Mg^2+^, and the reaction is non-reversible at the 3′-5′ exonuclease active site.

Previously reported qualitative data on 9°N polD showed that 3′-5′ exonuclease activity was dependent on Mg^2+^ or Mn^2+^, with an increase in activity seen for Mn^2+^ (20). The quantitative pre-steady-state exonuclease kinetics performed here reveal a ≥44-fold increase in *k*_exo_ in the presence of Mn^2+^ *versus* Mg^2+^ (Table 2). Similar to 9°N polD, the 3′-5′ exonuclease activity of polD from *Pyrococcus horikoshii* is more robust in the presence of Mn^2+^ over Mg^2+^, whereas polD from *Methanococcus jannaschii* requires Mn^2+^ and is inactive with Mg^2+^ (13, 33, 34). Differences in metal requirements between species suggest that the 3′-5′ exonuclease active site of the polD small subunit may differ between different archaeal species.

The polD 3′-5′ exonuclease small subunit bears homology to the calcineurin-like phosphoesterase family (35–37). This calcineurin-like phosphoesterase superfamily requires the presence of two divalent metal ions per active site for catalysis, typically Mn^2+^, Ni^2+^, Ca^2+^, Fe^2+^, Fe^3+^, or Zn^2+^, and contains a wide array of members, including phosphoserine/threonine phosphodiesterases, nucleotidases, and nucleases (38). The polD homology with calcineurin-like phosphoesterases and its preference for Mn^2+^ over Mg^2+^ suggest that the polD small subunit may have been an independently active exonuclease enzyme. Other archaeal replisome proteins, including the GINS-associated nuclease (GAN), a ssDNA 5′-3′ exonuclease, rely upon Mn^2+^ for exonuclease activity, with limited activity observed in the presence of Mg^2+^ (17). Together, these data suggest a putative role of Mn^2+^ as a cofactor during archaeal replication. However, it is still unclear which metal ion, Mn^2+^ or Mg^2+^, is bound within the polD 3′-5′ exonuclease site *in vivo*. The *in vivo* concentrations of Mn^2+^ and Mg^2+^ as well as the relative binding affinities of the two metals in the 3′-5′ exonuclease active site (both unknown parameters) determine which metal is bound *in vivo*.

##### Comparison of Nucleotide Selectivity among Different Polymerase Families

Polymerases have evolved highly specific mechanisms to ensure selection and incorporation of the correct nucleotide. The pre-steady-state nucleotide incorporation kinetics performed in this work confirm the presence of nucleotide discrimination mechanisms within polD and suggest that discrimination is achieved through weak binding and slow catalysis of incorrect nucleotides. Due to the lack of polD structural characterization studies, it is not yet possible to easily identify active site determinants for nucleotide discrimination.

A quantitative assessment of incorrect nucleotide incorporation is reflected by nucleotide selectivity constants. Polymerases with high nucleotide selectivity are less likely to incorporate incorrect nucleotides and are therefore less likely to introduce mutations. Therefore, high nucleotide selectivity (incorporation of correct *versus* incorrect dNTP) is an important polymerase feature to ensure accurate genome replication. Table 6 compares the average nucleotide selectivity of several DNA polymerase families. Selectivity values range from as high as 2.7 × 10^5^ in the replicative Family B RB69 DNA polymerase (39) to as low as 3.0 × 10^3^ in the translesion Family Y Dpo4 DNA polymerase (40). polD nucleotide selectivity is relatively low compared with other families, especially for a polymerase implicated in playing a replicative role, with an average nucleotide selectivity of 4.3 × 10^3^. Likewise, the data obtained here correspond well to previously published fidelity data, which suggests that polD has a higher error rate than typical replicative polymerases (41). We suspect the low fidelity observed for 9°N polD is due to the slow rate of polymerization observed for correct nucleotide incorporation, *k*_pol_ between 1.8 and 3.1 s^−1^, in conjunction with the small decrease observed in *k*_pol_ from correct to incorrect nucleotide incorporation (from 1.5- to 45-fold). Although the large increase in *K_d_*_(dNTP)_ from correct to incorrect nucleotide binding in 9°N polD (from 120- to 1500-fold) somewhat offsets the small change in *k*_pol_, replicative polymerases typically have a significant -fold change in both *k*_pol_ and *K_d_*_(dNTP)_ during nucleotide discrimination. It is possible, and quite likely, that the *k*_pol_ and, consequently, the nucleotide selectivity and fidelity of polD are highly dependent upon the presence of replisome components, such as proliferating cell nuclear antigen, which are absent in our *in vitro* studies.

**TABLE 6:** **Comparison of the average nucleotide selectivity for polymerase families A, B, D, X, and Y**

Polymerase	Family	*k*_pol_/*K_d_* correct	*k*_pol_/*K_d_* incorrect	Nucleotide selectivity*^a^*
		μ*m*^−*1*^ *s*^−*1*^	μ*m*^−*1*^ *s*^−*1*^	
KF (21)	A	11	1.2 × 10^−3^	4.1 × 10^4^
RB69 (39)	B	2.9	1.1 × 10^−5^	2.7 × 10^5^
9°N PolD (this work)	D	1.6	6.1 × 10^−4^	4.3 × 10^3^
rPolβ (23)	X	5.0	5.9 × 10^−4^	3.3 × 10^4^
Dpo4 (40)	Y	7.0 × 10^−2^	3.3 × 10^−5^	3.0 × 10^3^

*^a^* Calculated from (*k*_pol_/*K_d_*) correct/(*k*_pol_/*K_d_*) incorrect.

##### Ribonucleotide Discrimination

In the cell, rNTPs are present in a 1000-fold excess over dNTPs (30). To cope with this imbalance and to maintain genome integrity during replication and repair, DNA polymerases have evolved specific mechanisms to exclude rNTPs (29). Family A, B, and Y DNA polymerases and RTs exclude rNTPs by a clash between a conserved bulky side chain “steric gate” amino acid and the rNTP C2′-OH. In Family X DNA polymerases (polλ and polβ), an active site backbone carbonyl clashes with the ribose C2′-OH, preventing rNTP incorporation (42, 43). These clashes prevent the binding and incorporation of the rNTP (25, 44–46). The kinetic basis of rNTP discrimination in Family A, B, and RT is due to weak binding (high *K_d_*) and slower incorporation (low *k*_pol_) compared with dNTPs. Family Y DNA polymerases bind rNTPs and dNTPs with similar affinity; therefore, discrimination occurs during catalysis (>6000-fold reduction in *k*_pol_ for rNTPs)(47, 48).

The kinetic analysis of rATP incorporation by polD performed here shows that discrimination is primarily due to weak rATP binding (144-fold higher *K_d_*) and slower incorporation (16-fold slower *k*_pol_) compared with dATP. Kinetic data are similar to Family A, B, Y, and RT discrimination kinetics and suggest that a polD active site amino acid may block rNTP. However, the amino acid(s) important for rNTP discrimination in polD are currently unknown. polD lacks conserved steric gate motifs (28, 29), and in the absence of polD structural information, the location and identity of steric gate amino acids for rNTP discrimination within the polD family remain elusive.

##### Conclusions and Future Directions

The kinetics performed here on polD (the most recent DNA polymerase family to be identified) allows a comprehensive and direct comparison of nucleotide incorporation and 3′-5′ exonuclease activities of all currently known DNA polymerase families. polD employs nucleotide discrimination mechanisms to prevent misincorporations and contains an active site steric gate amino acid to prevent ribonucleotide incorporation, similar to other DNA polymerase families. Due to a lack of polD structural information, the identity of nucleotide discrimination and steric gate active site determinants remains unclear and will be the focus of future structural studies. Furthermore, structural data will help to identify the polD active site, needed to explore the proposed conformational changes associated with nucleotide incorporation, including open and closed polymerase conformations. Although polD has been implicated as the major replicative polymerase in much of Archaea, the kinetic work performed here suggests low polymerase fidelity. We propose that the presence of other replisome components, such as proliferating cell nuclear antigen, will increase polD fidelity. The effect of these replisome components on polD fidelity will be explored in future work. Finally, although polD contains a 3′-5′ exonuclease hydrolysis activity similar to many other polymerase families, the reliance on Mn^2+^ for optimal activity is unique to the polD family and importantly suggests that Mn^2+^ may play an important role in polD fidelity. Our data demonstrate that polD follows the same overall kinetic pathway as the other DNA polymerase families despite being a two-subunit enzyme with little sequence similarity to other families of DNA polymerases. Importantly, despite active site divergence among families, DNA polymerases, including polD, have each evolved specific mechanisms to accurately and faithfully replicate genomes.

## Author Contributions

K. M. S. and A. F. G. designed research. K. M. S. performed research. K. M. S. and A. F. G. analyzed data and wrote and approved the final version of the manuscript.

## Footnotes

2 The abbreviations used are:

polD: Family D DNA polymerase
FAM: 5-carboxyfluorescein
nt: nucleotide(s)
RQF: rapid chemical quench instrument.
